# Dysregulation of RNA Binding Protein Aggregation in Neurodegenerative Disorders

**DOI:** 10.3389/fnmol.2017.00089

**Published:** 2017-04-04

**Authors:** Brandon Maziuk, Heather I. Ballance, Benjamin Wolozin

**Affiliations:** Department of Pharmacology and Neurology, Boston University School of MedicineBoston, MA, USA

**Keywords:** RNA metabolism, RNA Translation, stress response, RNA binding proteins, tau aggregation

## Abstract

The unique biology of RNA binding proteins is altering our view of the genesis of protein misfolding diseases. These proteins use aggregation of low complexity domains (LCDs) as a means to regulate the localization and utilization of RNA by forming RNA granules, such as stress granules, transport granules and P-bodies. The reliance on reversible aggregation as a mechanism for biological regulation renders this family of proteins highly vulnerable to promoting diseases of protein misfolding. Mutations in RNA binding proteins are associated with many neurodegenerative disorders, such as amyotrophic lateral sclerosis (ALS) and frontotemporal lobar dementia (FTLD). The biology of RNA binding proteins also extends to microtubule associated protein tau. Tau is normally an axonal protein, but in stress it translocates to the somatodendritic arbor where it takes on a new function promoting formation of stress granules. The interaction of tau with stress granules also promotes tau aggregation, accelerating formation of the tau pathology that we associate with diseases such as Alzheimer's disease (AD).

## Introduction

Pathological protein aggregation is a hallmark of neurodegenerative disorders such as Alzheimer's Disease (AD), Parkinson's Disease (PD), Frontotemporal lobar dementia (FTLD), and Amyotrophic Lateral Sclerosis (ALS). Classically, this aggregation has been viewed as the byproduct of protein misfolding and/or impairment of protein catabolism. In this model, misfolded proteins and protein oligomers accumulate because of increased production or reduced removal. The amount of misfolded protein and oligomer is thought to depend primarily on the initial amount of starting protein and its natural propensity to aggregate. The accumulating misfolded proteins and oligomers lead to the uncoordinated assembly of higher order oligomers, amyloids and eventually large fibrils, which harm the cell and precipitate neurodegeneration.

Over the past decade an increasing body of work investigating the unusual biology of RNA binding proteins (RBPs) has shed new insight into mechanisms of protein aggregation in disease (Li et al., 2013). RBPs represent a class of over 800 proteins responsible for the regulation of mRNA maturation in the nucleus as well as mRNA translation in the cytoplasm. The domain structure of these proteins typically includes RNA recognition motifs, aggregation promoting low complexity domains (LCDs), and nuclear import and export sequences (Lunde et al., 2007; King et al., 2012; Espinosa Angarica et al., 2014). Importantly, RBPs form a variety of RNA-protein (RNP) granules that are critical for RNA metabolism; these granules include processing-bodies (P-bodies), stress granules (SGs), nuclear granules, and/or transport granules (Kedersha et al., 1999). P-bodies are responsible for mRNA silencing and degradation, while SGs function to suppress the translation of non-essential proteins in favor of protective stress-response proteins. SGs are nucleated by a core set of RBPs (see below) with their associated transcripts, and maturation of the SG incorporates secondary RBPs, additional transcripts, and proteins that enable interactions with other organelles, such as microtubules, actin filaments, autophagosomes and mitochondria (Buchan and Parker, 2009; Vanderweyde et al., 2016).

RBPs share important genetic and pathological links with neurological diseases (Table 1). Tar DNA binding protein (gene, TARDBP; protein, TDP-43) is the primary component of pathological aggregates in most cases of ALS as well as the 40% of cases of FTLD associated with progranulin haplo-insufficiency; in addition, mutations in TDP-43 proteins cause familial ALS. As described in Table 1, mutations in FUS, hnRNPA1/B2 and other RBPs are associated with familial forms of motor neuron disorders, while mutations in proteins associated with formation or removal of RNA granules cause ALS, FTLD, other motor neuron diseases as well as myopathies. Recent studies from our laboratory also implicate RBPs such as TIA1 in the pathophysiology of AD and other tauopathies (Vanderweyde et al., 2012, 2016). Histopathological studies also implicate RBPs in Huntington's Disease (HD), and Creutzfeldt-Jakob Disease (CJD). These discoveries have propelled extensive efforts to understand how dysregulation of RBP aggregation leads to neurodegenerative conditions.

**Table 1:** **RNA binding proteins implicated in neurodegenerative disorders**.

**RNA binding protein**	**Abbreviation**	**Associated diseases**
TAR DNA-binding protein 43	TDP43	ALS^*^^∧^, FTLD^*^^∧^, AD^∧^, HD^∧^
T-cell intracellular antigen 1	TIA1	ALS^∧^, FTLD^∧^, AD^∧^
Ras GTPase-activating protein-binding protein 1	G3BP1	ALS^∧^, FTLD^∧^, AD^∧^
Tristetraprolin	TTP	ALS^∧^, FTLD^∧^, AD^∧^
Fused in Sarcoma	FUS	ALS^*^^∧^, FTLD^*^^∧^
Ewing Sarcoma Protein	EWS	ALS^*^^∧^, FTLD^*^^∧^
TATA-Box Binding Protein Associated Factor 15	TAF15	ALS^*^^∧^, FTLD^*^^∧^
Heterogeneous Ribonucleoprotein Particle A1/A2	hnRNPA1/A2	ALS^*^, FTLD^*^
Angiogenin	ANG	ALS, PD^*^
Survival of motor neuron	SMN1	ALS^*^, SMA^*^
Matrin-3	MATR3	ALS^∧^
Ataxin-2	ATXN2	ALS^∧^
Optineurin	OPTN	ALS^*^^∧^
Fragile X mental retardation protein	FMRP	FXS^*^

* *Mutations linked to disease*.

∧ *Inclusions linked to disease*.

*ALS, Amyotrophic Lateral Sclerosis; FTLD, Frontotemporal Lobar Dementia; AD, Alzheimer's Disease; HD, Huntington's Disease; PD, Parkinson's Disease; FSX, Fragile X Syndrome; SMA, Spinal Muscular Atrophy*.

## RNP granules and their relevance to neurodegenerative disorders

### Stress granules and P-bodies

SGs and P-bodies are key RNP granules that transiently consolidate cytoplasmic mRNAs. SGs form rapidly with cellular stress; stress kinases phosphorylate translation initiation factor eIF2α, which promotes polysome disassembly and SG formation (Kedersha et al., 1999). Some of the core SG nucleating RBPs include TIA1 (T-cell intracellular antigen 1), nucleolysin (TIAR), tristetraprolin (TTP), fragile X mental retardation protein (FMRP) and Ras-GAP SH3 Binding Protein 1 (G3BP1). Aggregation of these core nucleating proteins to form SGs initiates a process in which mRNAs stalled in translation are sequestered, allowing RNA translation to shift toward synthesis of cytoprotective proteins (Kedersha and Anderson, 2007). P-bodies associate with SGs and exchange transcripts which are subject to decapping and degradation (for detailed review on RNP granule components and dynamics, see Protter and Parker, 2016).

RNP granules are dynamic, diverse structures, containing a variety of RBPs, enzymes, remodeling proteins, and transcripts (Protter and Parker, 2016). The nucleating components of RNP granules can differ based on environmental conditions, the mechanisms controlling granule formation, and cell type. SGs also evolve with time, moving from small primary stress granules to larger secondary stress granules (McDonald et al., 2011); further evolution might occur as mature stress granules become persistent pathological stress granules over the months to years associated with human disease.

Initial evidence implicating stress granules in neurodegenerative diseases arose from the seminal discovery that TDP-43 is the primary pathological protein that accumulates in sporadic ALS and many cases of FTD (Neumann et al., 2006). Following this discovery, ALS-linked mutations were identified in multiple RBPs, including TDP-43, FUS, ATXN2, hnRNPA1, EWSR1, and TAF15 (Table 1). The disease-linked mutations generally occur in the low complexity (LC) domains of these proteins and increase the tendency of the proteins to aggregate, which will be discussed in a later section (Johnson et al., 2009; Liu-Yesucevitz et al., 2010; Kim et al., 2013); mutations in other ALS-linked genes also stabilize stress granule dynamics, including mutations in C9orf72, Valosin containing protein (VCP) and Cu/Zn Superoxide Dismutase (SOD1) (Buchan et al., 2013; Gal et al., 2016; Lee et al., 2016; Lin et al., 2016).

### Neuronal transport RNP granules

Neurons have a unique need for controlled transport of mRNAs because of their lengthy processes and their need for activity dependent translation at the synapse. These needs demand high expression of RNP transport granules in the somatodendritic arbor. Transport granules contain RBPs such as staufen, pamillo and FMRP; these granules play important roles in mRNA localization and activity-dependent translational control at the synapse (Protter and Parker, 2016). Transport granules might represent the sites where RBP dysfunction in disease is first evident. Studies of granule trafficking in living neurons show that disease-linked mutations in RBPs, such as TDP-43, produce granules that are innately larger and travel slower (Liu-Yesucevitz et al., 2014). These larger, slower moving granules might be a nidus for disease pathology, which could provide one mechanism through which RBPs primarily might cause neuronal disease.

### Nuclear RNP granules

The nucleus is highly enriched for many RBPs responsible for transcription and early RNA maturation events such as splicing, capping, or nuclear export. A variety of RNP granules organize these many critical functions, including coiled bodies, PML bodies, Cajal bodies, nucleoli, speckles, gems, and histone locus bodies (HLBs), but how nuclear RNP granule deficiency contributes to neurodegenerative disorders is poorly understood. Disease linked RBPs including FUS, EWS and TAF15 mediate a DNA damage response through the formation of an RNP granule around DNA breaks (Wang et al., 2013; Deng et al., 2014). Dipeptide repeats produced by mutations in C9orf72 might also interfere with multiple nuclear functions (see below). In addition, RBP45 is an RBP present in ALS, FTLD and AD inclusions that is associated with nuclear splicing pathways (Li et al., 2016), while transcriptomic studies from ALS patients have revealed trends in RNA editing errors and disease related differences in splicing alterations (Prudencio et al., 2015). Crosslinked immunoprecipitation (iCLIP) studies of TDP-43 and FUS indicate that TDP-43 exhibits a preference for binding long transcripts of neuron enriched proteins, and FUS appears to function as a marker for RNA polymerase elongation (Polymenidou et al., 2011, 2012). These studies highlight a potentially important role for nuclear RNA metabolism in ALS.

### C9orf72 and RNA foci in ALS

Hexanucleotide repeat expansions in a non-coding region of the gene *C9orf72* (C9) produce pleotropic pathology that are a major cause of familial ALS, and also contribute to other neurological diseases. This mutant expansion was initially identified and found to induce the formation of RNA foci from the sense strand of the C9 mRNA (DeJesus-Hernandez et al., 2011; Renton et al., 2011). Mutant C9 also produces a non-canonical form of translation generating 6 different dipeptide repeats (DPRs) that accumulate as aggregates in diseased brains (Ash et al., 2013; Mori et al., 2013a). C9 pathology also exhibits nuclear to cytoplasmic translocation of TDP-43 and formation of TDP-43 inclusions, pathologies which cause many problems (Lee et al., 2012). RNA foci resulting from mutant C9 transcripts recruit a large variety of RBPs (Mori et al., 2013b), and DPRs appear to interfere with multiple functions, including those of the nucleolus, the nuclear pore and other RBPs (Kwon et al., 2014; Jovicic et al., 2015; Lee et al., 2016; Lin et al., 2016). C9orf72 repeats have been shown to affect stress granule formation in cell N2A cells and in cortical neurons using the response to protein synthesis inhibitors as a marker of stress granule function. The protein synthesis inhibitor puromycin allows the ribosome to run-off the mRNA, causing the accumulation of naked mRNA, which promotes stress granule formation; the protein synthesis inhibitor cycloheximide stalls ribosomes on mRNA, which hides the mRNA and inhibits stress granule formation. C9orf72 repeat foci co-localize with TIA1 puncta in the cytoplasm in response to puromycin, but not in response to cycloheximide (Maharjan et al., 2016). Conversely, CRISPR mediated deletion of C9 inhibits stress granule formation (Maharjan et al., 2016), while overexpressing C9 leads to stress granule formation in the absence of stressors. For a detailed review of the evolving research into the pathophysiology of C9 disease see the review by Todd and Petrucelli.

## How do RNP granules transition from a healthy to pathological state?

### Phase separation as the first step to fibrillization

A key feature of many RBP sequences is the presence of a “prion-like” LCD enriched in uncharged polar amino acids (King et al., 2012). Typically, these sequences are at least 60 amino acids longs, are predicted to be intrinsically unfolded, and enable the replication of a particular protein conformation from one copy to another (Couthouis et al., 2011). The LCDss are also conducive to mathematical modeling based on glutamine and asparagine enrichment, and such modeling has shown that many of the disease-linked RBPs are those which have the greatest tendency to aggregate (Michelitsch and Weissman, 2000; Alberti et al., 2009; Zambrano et al., 2015).

**Figure 1:**
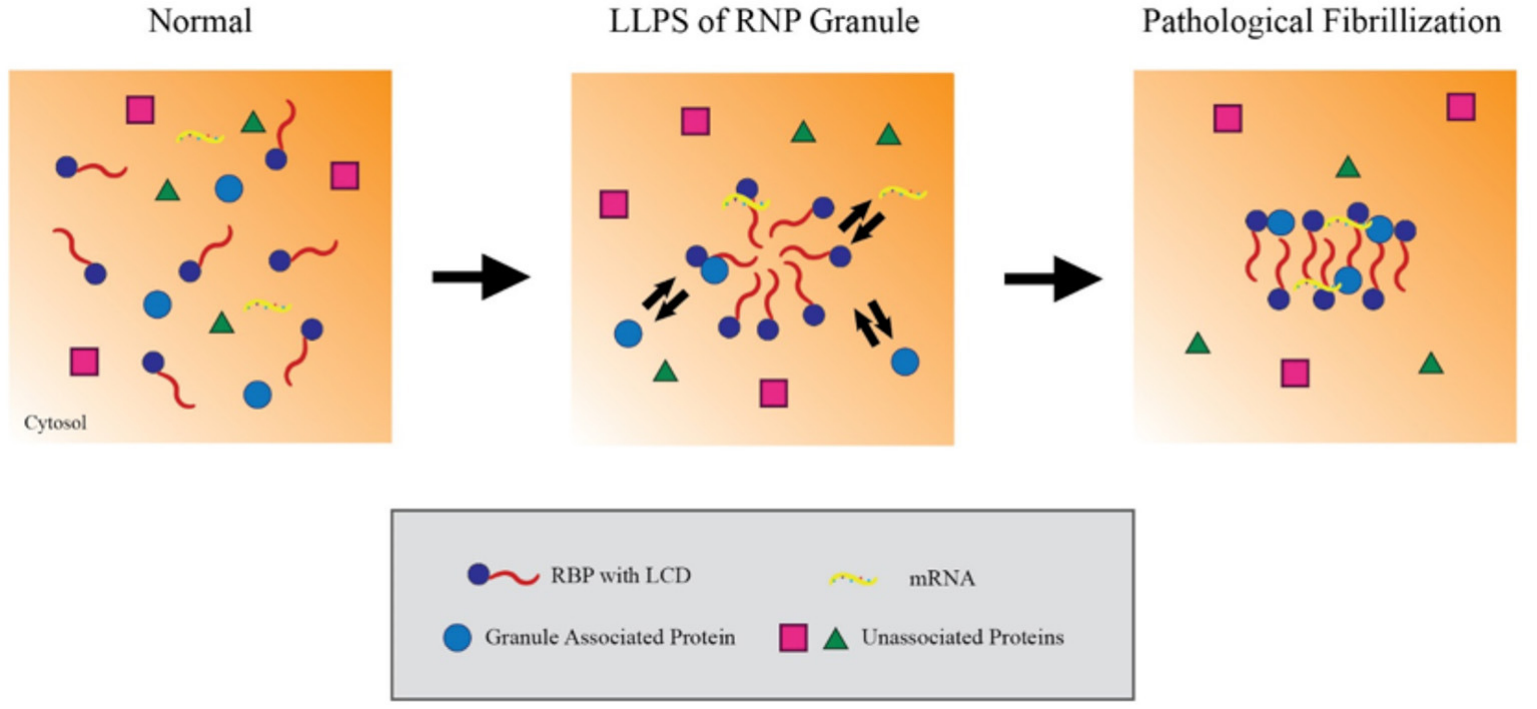
**Liquid-liquid phase separation contributes to RNP granule dynamics and eventual fibrillization**. Within the cytosol, RNA binding proteins (RBPs) containing low complexity prion-like domains (LCDs) exist in an unaggregated state. Upon activating signaling cascades, the LCDs mediate weak aggregation through liquid-liquid phase separation, creating a distinct compartment enriched in RBPs, RNA and other associated proteins. Importantly, these ribonucleoprotein granules maintain the ability to exchange material with the cytosol while in this state, which allows them to carry out key mRNA regulating events. However, persistent aggregation over time or other pathological insults can drive these RBPs to further aggregate into compact, stable fibrils. In this model, the fibrillar forms of these granules can no longer exchange material with the surrounding cytosol and effectively trap their components in the granule.

RBPs appear to form RNP granules through a process of liquid-liquid phase separation (LLPS) mediated by their LCDss (Lin et al., 2015). LLPS refers to a biochemical process which forms distinct, non-membrane bound complexes within the cytoplasm that behave in a manner analogous to oil droplets in an aqueous solution. LLPS is hypothesized to allow RNA granules to be particularly dynamic, being sufficiently stable to facilitate processes such as RNA transport but also sufficiently dynamic to interact with the surrounding cytosol and readily disperse when acted on by cellular disaggregases (e.g., VCP, Hsp110 or DNAJB1), chaperones (e.g., FKBPs) or signaling mechanisms. Studies using recombinant proteins or cell lines indicate that the RBPs hnRNPA1 and FUS cycle through LLPS in a process that is dependent on the C-terminal LCDss (Figure 1; Molliex et al., 2015). Upon repetitive cycling, a small fraction of the protein misfolds to form highly stable amyloids; disease-linked mutations in these proteins strongly increase the likelihood to form such amyloids, perhaps providing the basis for the persistent pathological inclusions that accumulate in ALS (Figure 1; Murakami et al., 2015; Patel et al., 2015). The biophysical mechanisms leading to TDP-43 pathology, though, are less clear (Johnson et al., 2009; Liu-Yesucevitz et al., 2010; Conicella et al., 2016). While TDP-43 readily forms stable aggregates and stable stress granules *in vitro* and in cultured neurons, recombinant forms of TDP-43 do not readily form hydrogels or undergo LLPS, and ALS-associated mutations in TDP-43 might even disrupt phase separation and self-interaction.

LLPS provides a compelling mechanistic link between the low complexity prion domains in disease related RBPs and the pathological aggregation of RBP nucleated granules. The simplicity of the experimental systems used for the studies described above provides powerful strengths as well as weaknesses. Use of purified recombinant proteins in *in vitro* studies allows rigorous analysis of the biophysical properties of the RBPs. Such studies might ultimately provide valuable insights into the mechanisms through which differing mutations can produce distinct disorders, such as is observed for the continuum of disease between ALS and FTD. However, these studies do not address two important issues. The first issue is the biological complexity of neurons, which have multiple levels of regulation provided by chaperones, post-translational modifications, RNA, disaggregates, the autolysosomal system, as well as the deleterious effects of aging. The second issue is the question of whether more aggregation is good or bad. Multiple different studies suggest that oligomers are more toxic to neurons than large inclusions, such as PrP, polyQ aggregates, Aβ plaques or neurofibrillary tangles (Arrasate et al., 2004; Santacruz et al., 2005; Silveira et al., 2005). For mechanisms of toxicity that are caused directly by aggregates, the accumulation of small oligomers might be more toxic than the accumulation of large inclusions. Conversely, large inclusions could be important for mechanisms of toxicity that arise loss of function due to sequestration of proteins, such as RBPs, in insoluble aggregates similar to stress granules or large nuclear inclusions. Thus, although LLPS provides an appealing mechanism to explain how aggregation might be initiated, care must be taken when considering how this paradigm might translate to the brain.

### Tau pathology promotes the formation of insoluble RBP aggregates

The microtubule associated protein tau is a multi-functional protein that is natively disordered, aggregation prone, and can disrupt protein homeostasis through several known mechanisms. Notably, it has been shown that increased levels of pathological tau lowers protein synthesis *in vitro* and *in vivo* (Meier et al., 2016). Over-expression of P301L mutant tau in rTg4510 mice upregulates the stress kinase PERK, leading to eIF2α phosphorylation, translation inhibition, and synapse loss (Meier et al., 2015; Vanderweyde et al., 2016). Inhibition of PERK ameliorates these deficits (Vanderweyde et al., 2016). Tau also affects protein translation through direct interaction with the ribosomes and RBPs. These interactions are both altered in AD, where association with RBPs is increased (Meier et al., 2016). Tau also modulates trafficking of RNA granules, with retrograde trafficking affected more than anterograde motion (Vanderweyde et al., 2016).

**Figure 2:**
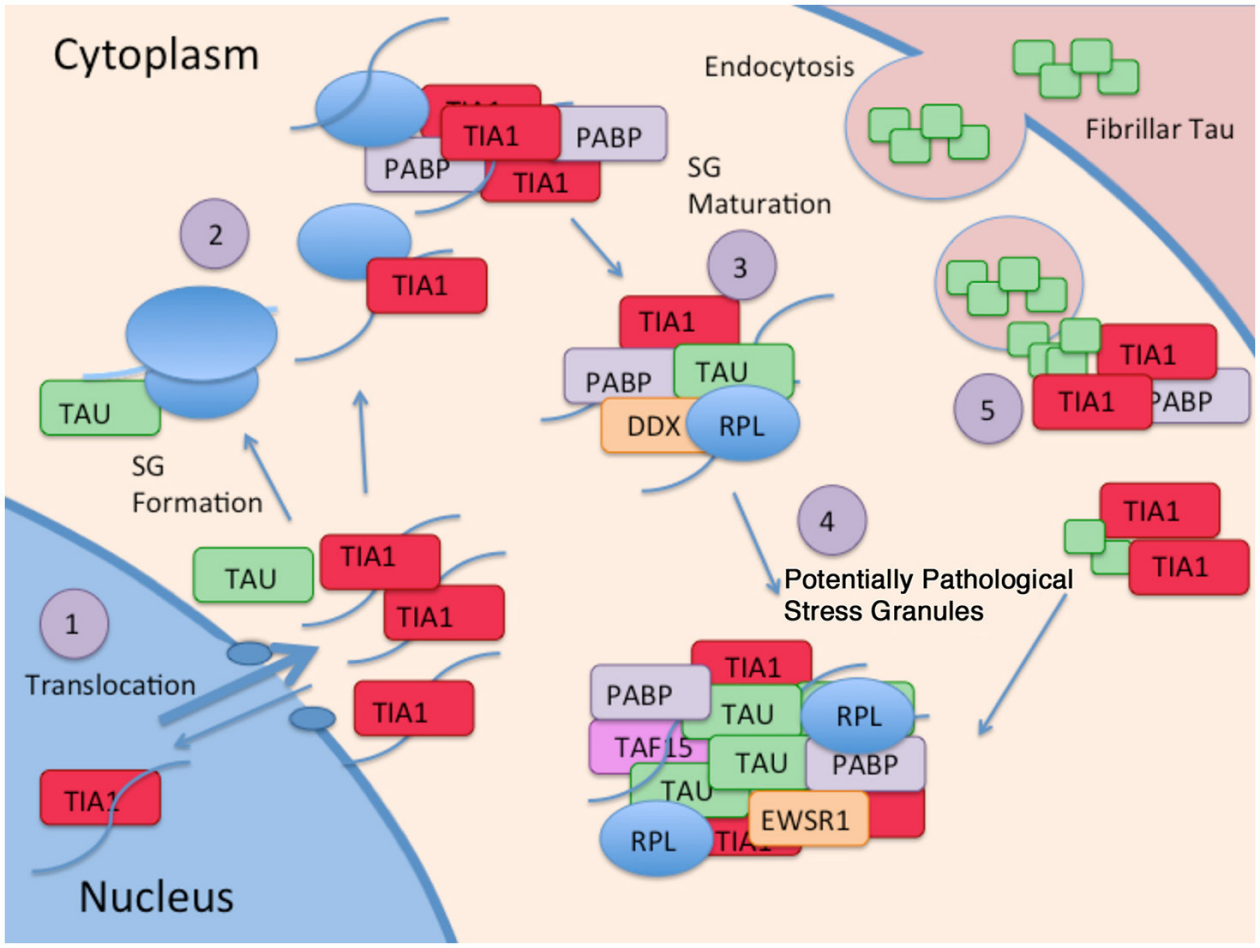
**Interaction of Tau and RBP pathologies: (1)** RBPs such as TIA1 splice RNA in the nucleus, and shuttle in and out of the nucleus. Although TIA1 is normally predominantly nuclear, Tau slows nuclear/cytoplasmic transport, decreasing anterograde transport less than retrograde transport, overall favoring a cytoplasmic localization of TIA1. **(2)** Tau binds to the small subunit of ribosomes, and this interaction changes in taupathies such as AD, resulting in stalled translation. Stalled translation initiation complexes containing mRNA and the small ribosomal subunit forms complexes with ribosomal proteins, mRNA, and core SG nucleating RBPs. **(3)** Small core stress granules forms complexes with additional RBPs including DDX helicases. **(4)** Tau increases the size of stress granules, and influences the specificity of RBPs included in stress granules. RBPs in complex with Tau and TIA1 include TAF15 and EWSR1. **(5)** TIA1 mediates interaction of stress granules with insoluble tau aggregates internalized from the cytoplasm (Meier et al., 2015; Brunello et al., 2016; Vanderweyde et al., 2016).

New evidence identifying a novel mechanism through which tau alters protein homeostasis indicates that tau interacts with TIA1, a core SG nucleating protein, and other RBPs (Figure 2). Tau pathology increases somatodendritic localization of TIA1, which is predominantly nuclear in healthy or unstressed cells (Vanderweyde et al., 2016). Tau/TIA1 complex formation accelerates the dynamics of SG formation, increases SG size, and concomitantly escalates tau aggregation. Disease-linked mutations enhance the effects of tau on SGs, with P301L tau generating TIA1 SGs that are larger but fewer in number than those formed in cells expressing WT tau (Vanderweyde et al., 2016). Extracellular tau aggregates also increase SG formation raising the possibility that tau propagation also contributes to formation of pathological SGs (Brunello et al., 2016). Tau also modifies the TIA1 interactome, where multiple RBPs including SNRNP70, EWSR1, TAF15, and several ribosomal proteins form complexes with TIA1 in a tau dependent manner (Vanderweyde et al., 2016). It is important to note that the effects of tau on SG dynamics are TIA1 specific; TDP-43 and FUS do not co-localize with tau, nor do the RBPs G3BP and TTP despite being core nucleating SG proteins (Vanderweyde et al., 2012). Thus, tau appears to selectively regulate SGs containing TIA1 as a core component (Figure 2).

The interaction of tau with SGs is a two way street that has important implications for disease mechanisms, and possibly for disease therapy. The interaction of TIA1 with tau increases the tendency of tau to form sarkosyl insoluble aggregates, and stabilizes tau dynamics (Vanderweyde et al., 2016). This means that the interaction of tau with SGs and perhaps other RNA granules might be previously undiscovered mechanism propelling tau aggregation and tau-mediated neurodegeneration (Figure 2). Conversely, knock down of TIA1 in primary hippocampal neurons reduces levels of misfolded tau, prevents acute toxicity associated with expressing P301L tau (Vanderweyde et al., 2016). The latter observation raises the possibility that reducing TIA1 might delay disease progression in tauopathies.

## Deregulation of stress granule removal contributes to disease

The dynamic nature of RBP aggregation to form SGs and other RNA granules provides a sharp contrast with the classic view of aggregation processes in neurodegeneration. RBP aggregation is readily reversible, and SGs formed by an acute stress also disperse just as rapidly following removal of stress (Protter and Parker, 2016). SG dispersal uses some of the same pathways responsible for clearing aggregated proteins, including heat shock protein chaperones, ubiquitin, p62/SQUSTM, proteasomal degradation and autophagy. Timely clearance of stress granules and interacting aggregating proteins such as tau and C9ORF72 are crucial for re-initiation of mRNA translation, as well as prevention of buildup of intrinsically disordered proteins that are prone to aggregation. Failure to clear stress granules, prion domain containing RBPs, and stress granule associated disordered proteins such as tau may lead to pathological protein aggregates (Wolozin, 2012; Protter and Parker, 2016).

### Heat shock proteins

Heat shock chaperones (HSPs) play a key role in responding to many of the same cell stressors that induce stress granules. While stress granules sequester non-essential mRNAs in the cytoplasm, heat shock transcripts are still translated. HSPs reduce protein misfolding and target existing misfolded/aggregated proteins for degradation, protecting neurons from injury caused by the accumulation of misfolded/aggregated proteins. Binding of HSPs to the LCDs of RBPs reduces their aggregation propensity; binding to the disordered domains of proteins such as tau or α-synuclein reduces the assembly of these aggregation prone regions as well. Hsp27 interacts directly with tau, specifically hyper-phosphorylated tau and PHFs (Abisambra et al., 2010). Studies with recombinant proteins and *in vivo* indicate that Hsp27, along with Hsp90 and other chaperones, promote maintenance of soluble tau, and reduction of tau fibrils (Abisambra et al., 2010). Hsp70 also enables tau solubility and tau interaction with microtubules (Abisambra et al., 2010). Whether tau association with stress granules and RBPs affects interaction with HSPs is unknown, but is an important outstanding question.

HSPs survey the proteome, and target misfolded proteins that must be degraded for ubiquitination. This process is crucial for clearance of misfolded tau, as aggregates containing both ubiquitin and tau occur in AD and FTLD-U. This surveillance function is mediated by the interaction of Hsp70 with CHIP, a E3 ubiquitin ligase; CHIP knockout mice exhibit increased tau pathology, while overexpression of CHIP has the opposite effect (Dickey et al., 2006). Ubiquitination becomes important in the SG cascade because it provides a major mechanisms through which persistent SGs can be targeted for clearance through autophagy and the UPS.

### Ubiquitin, autophagy, and stress granules

The importance of clearing protein aggregates to neurodegenerative diseases is apparent in protein catabolism genes linked to FTD, ALS, or myopathies, and stress granules are also disassembled by components of the proteasome and autophagy/lysosmal degradation pathways (Buchan et al., 2013). Mutations in SQSTM1/p62, ubiquilin-2 and VCP are all associated with familial forms of ALS, FTD or myopathy (Deng et al., 2011; Rubino et al., 2012). VCP is a disaggregase that functions in SG clearance, although it is a complicated molecule with many different activities (Buchan et al., 2013). P62/SQSTM1 and ubiquilin-2 both function in identifying pathological aggregates, such as persistent pathological SGs, and targeting them for removal. Mutations in SQSTM1 likely interfere with its function in targeting ubiquitinated proteins, including tau, for proteasomal degradation. Other genes linked to ALS and FTD, such as Tmem106b, Chmp2b, Tbk1, and Optineurin, have functions as adapter proteins that recognize ubiquitin tagged proteins. GRN, Tmem106B, and Chmp2b are also necessary for lysosmal function in autophagy.

## Targeting stress granule formation in neurodegenerative diseases

### Stress kinase activation of eIF2α

SG formation can be triggered by a variety of cellular stressors including heat or cold shock, osmotic shock, nutrient deprivation, ROS, or the unfolded protein responses (UPR) (Radford et al., 2015). These stresses activate stress kinases, which phosphorylate 40S-eukaryotic initiation factor 2 (eIF2α) and prevent further translation initiation. Non-phosphorylated eIF2α normally forms complexes with methionine tRNA and GTP as part of the RNA translation initiation complex (Kedersha et al., 1999). However, phosphorylation of eIF2α increases binding of eIF2α to eIF2B, which prevents the exchange of GDP for GTP and inhibits translation initiation (Kedersha et al., 1999). Phosphorylation of eIF2α is reversible through Growth Arrest and DNA Damage-inducible Protein 34 (GADD34), which is an adapter protein that recruits phosphatase Protein Phosphatase 1 (PP1). Dephosphorylation of eIF2α stimulates SG disassembly, which allows re-initiation of normal translation (Kedersha et al., 1999).

There are four core stress activated kinases that phosphorylate eIF2α: Protein Kinase R (PKR), PKR-like/Pancreatic Endoplasmic Reticulum kinase (PERK), Heme-Regulated Inhibitor (HRI), or General Control Non-derepressible 2 (GCN) (Taniuchi et al., 2016). PKR is activated by double stranded RNA, enabling it to respond to viral infection (Taniuchi et al., 2016). PKR appears to respond to viral activation of the unfolded protein response (UPR), and perhaps because of this, PKR also responds to the presence of aggregated Aβ and PRNP (Chang et al., 2002; Goggin et al., 2008). PERK also responds to the accumulation of misfolded or aggregated proteins as part of the UPR. PERK is activated in multiple different models of neurodegeneration, including those caused by overexpressing PrP, TDP-43, and tau (Moreno et al., 2013; Kim et al., 2014; Radford et al., 2015). PERK inhibition by inhibitors such as GSK20606414 restores translation and protects against degeneration in these models (Kim et al., 2014; Radford et al., 2015). Unfortunately, the clinical utility of PKR inhibitors might be limited by the risk that they will allow activation of latent viruses in elderly patients, while PERK inhibitors cause severe pancreatic toxicity (Yu et al., 2015). GCN2 is activated by nutrient deprivation, specifically the absence of essential amino acids, and by activation of the ubiquitin-proteasome system (UPS). GCN2 deletion protects against memory loss in a APP/PS1 mouse model, but not in a 5xFAD APP mouse model (Devi and Ohno, 2013; Ma et al., 2013). HRI is activated by oxidative stress induced by osmotic, heat shock, or arsenite, but has yet to be studied directly in neurodegeneration (Lu et al., 2001).

### eIF2α-independent inhibition of translation initiation

Translation can also be stalled through eIF2α independent mechanisms acting directly on components of the ribosome, particularly the small subunit involved in preinitation complexes (Kedersha and Anderson, 2007). Activated eIF2α binds to mRNA, but needs to interact with the eIF4F complex for initiation of translation. The eIF4F complex consists of eIF4A, B, E and G, which bind mRNA through the RBP poly-A binding protein (PABP). The role of the eIF4F complex in neurodegeneration has yet to be studied in detail, but its potential importance is evident when considering angiogenin, a protein with mutations associated with ALS and PD (van Es et al., 2011; Pan et al., 2015). The ribonuclease angiogenin cleaves tRNAs, to produce tiRNAs (tRNA-derived stress induced fragments). The 5' ends of tiRNAs displace components of the pre-initiation complex including eIF4G and eIF4A from mRNA, and displace eIF4F from the m^7^G mRNA cap (Ivanov et al., 2011). Mutations in eIF4G1 are weakly associated with familial PD; in this case the mechanism might lie in inappropriate aggregation with α-synuclein, potentially leading to either a loss of eIF4F function or a gain of α-synuclein aggregation (Siitonen et al., 2013; Dhungel et al., 2015). These independent lines of genetic evidence suggest a role for the eIF4F complex in the pathophysiology of neurodegenerative disease.

### Stalled translation initiation complexes

Stalling of translation through eIF2α dependent or independent pathways allows for disassembly of polyribosomes. The determinant of SG formation lies in whether the ribosomes remain associated with the transcripts. Stalling caused by the protein synthesis inhibitor puromycin disassociates the ribosome, producing naked transcript and initiating SG assembly (Kedersha et al., 1999). In contrast, treatment with the protein synthesis inhibitor cycloheximide leaves ribosomes associated with transcripts and prevents SG formation (Kedersha et al., 1999). Stalled translation initiation complexes contain components of the 40S small ribosomal subunit, including eIF4E, eIF4G, and eIF3, as well as PABP, a RBP that links eIF4 proteins to the mRNA. SGs form when the stalled complex is bound by core RBPs, such as TIA1, TIAR, TTP, or G3BP. The LC domains of these proteins are necessary for SG formation, likely because of the tendency of these LC domains to aggregate as described. Mutant TIA1 lacking an RNA binding domain inhibits the formation of stress granules, demonstrating the necessity of RNA binding for the formation of stress granules (Kedersha et al., 1999). As the SG matures, other RBPs are recruited, leading to the growth of the SG and the binding of a wider array of transcripts.

## Conclusion

RNA binding proteins have a rich biology that integrates well with our knowledge of the pathophysiology of neurodegenerative diseases. The core biology derives from the presence of LC domains that undergo LLPS, allowing for reversible aggregation and sequestration of transcripts and other binding proteins. This normally transient biological phenomenon appears to become persistent and pathological in disease, resulting in the interference the normal functions of RBPs and transcripts through excessive segregation. Emerging evidence from several laboratories demonstrate that tau protein contributes to SG biology by promoting SG aggregation in a process that leads to tau concomitant aggregation. In chronic disease, these aggregated proteins become persistent and pathological, which ultimately leads to the accumulation of aggregated protein, disease pathology and neurodegeneration. Fortunately, SG biology is regulated at multiple levels by a variety of pathways, which provides entirely new targets of pharmacotherapy for many neurodegenerative diseases, including ALS and AD.

## Author contributions

BM, HB, and BW all contributed to writing and editing the manuscript.

### Conflict of interest statement

BW is co-founder and Chief Scientific Officer of the biotechnology company Aquinnah Pharmaceuticals Inc. The other authors declare that the research was conducted in the absence of any commercial or financial relationships that could be construed as a potential conflict of interest.
